# Can Gaming Increase Antibiotic Awareness in Children? A Mixed-Methods Approach

**DOI:** 10.2196/games.6420

**Published:** 2017-03-24

**Authors:** Alexander R Hale, Vicki Louise Young, Ann Grand, Cliodna Ann Miriam McNulty

**Affiliations:** ^1^ Science Communication Unit University of the West of England Bristol United Kingdom; ^2^ Public Health England Primary Care Unit Gloucester United Kingdom; ^3^ School of Biological Sciences The University of Western Australia Crawley Australia

**Keywords:** antibiotic resistance, computer games, children, education, e-Bug

## Abstract

**Background:**

e-Bug is a pan-European educational resource for junior and senior school children, which contains activities covering prudent antibiotic use and the spread, treatment, and prevention of infection. Teaching resources for children aged 7-15 years are complemented by a student website that hosts games and interactive activities for the children to continue their learning at home.

**Objective:**

The aim of this study was to appraise young people’s opinions of 3 antibiotic games on the e-Bug student website, exploring children’s views and suggestions for improvements, and analyzing change in their knowledge around the learning outcomes covered. The 3 games selected for evaluation all contained elements and learning outcomes relating to antibiotics, the correct use of antibiotics, and bacteria and viruses.

**Methods:**

A mixed methodological approach was undertaken, wherein 153 pupils aged 9-11 years in primary schools and summer schools in the Bristol and Gloucestershire area completed a questionnaire with antibiotic and microbe questions, before and after playing 3 e-Bug games for a total of 15 minutes. The after questionnaire also contained open-ended and Likert scale questions. In addition, 6 focus groups with 48 students and think-aloud sessions with 4 students who had all played the games were performed.

**Results:**

The questionnaire data showed a significant increase in knowledge for 2 out of 7 questions (*P*=.01 and *P*<.001), whereas all questions showed a small level of increase. The two areas of significant knowledge improvement focused around the use of antibiotics for bacterial versus viral infections and ensuring the course of antibiotics is completed. Qualitative data showed that the e-Bug game “Body Busters” was the most popular, closely followed by “Doctor Doctor,” and “Microbe Mania” the least popular.

**Conclusions:**

This study shows that 2 of the e-Bug antibiotic educational games are valuable. “Body Busters” effectively increased antibiotic knowledge in children and had the greatest flow and enjoyment. “Doctor Doctor” also resulted in increased knowledge, but was less enjoyable. “Microbe Mania” had neither flow nor knowledge gain and therefore needs much modification and review. The results from the qualitative part of this study will be very important to inform future modifications and improvements to the e-Bug games.

## Introduction

There has been a continuous rise in the number of infections caused by antibiotic resistant bacteria, which, in part, has been driven by misuse of antibiotics by prescribers and patients [1-4]. The World Health Organization (WHO) has stated that antibiotic resistance has the potential to affect anyone [5], and therefore is a serious and growing problem that has been labeled as “a ticking time bomb, not only for the United Kingdom but also for the world” [6]. The National Institute of Clinical Excellence has advised that schools should teach all ages about prevention of infections, self-care, and antibiotic use [7].

The e-Bug project is a pan-European initiative that aims to help “reduce the incidence of antibiotic resistance across Europe by educating future prescribers and users on prudent antibiotic use” [8]. The e-Bug website contains 2 sections, junior and senior, aimed at pupils aged 9-11 years and 12-15 years respectively. These include downloadable classroom lesson plans for teachers and fact files, quizzes, and revision guides for students. The e-Bug website also hosts several games, to be used at school or in the home, aimed at improving young people’s understanding of respiratory and hand hygiene, and responsible antibiotic use [9,10].

Since the 1970s, the global market for computer games has grown exponentially and games have advanced in terms of complexity, graphics, interaction, and narrative; attracting an ever-increasing number of gamers. Different theories explain how and why these games are so popular and can immerse a player for extended periods. Csikszentmihalyi theorized “flow” [11], which he described as “the state in which people are so involved in an activity that nothing else matters” [12]. The flow state has particular characteristics, including intense concentration, a sense of control of the situation, an altered personal experience of time, and a loss of reflective self-consciousness [13]. To access the flow state, an activity such as playing a computer game needs to create a balance between the difficulty of the challenge and the skills of the player [14].

In recent years, Web-based tools have been increasingly used in schools and other settings for education. Many studies have looked at the effectiveness of serious games, particularly those that cover health topics. A meta-analysis showed that serious games have a small but positive effect on healthy lifestyles and knowledge improvement [15]. Serious games are defined as games which have a primary goal of education, rather than entertainment [16].

Many educational games focus around health and science subjects. In public health, these include smoking cessation [17] and diet and exercise [18]. Web-based tools such as Twitter and social media have also been used to raise awareness and educate about antibiotics [19,20]. An antibiotic game to educate clinicians exists [21], but no material has been published on the development or evaluation of Web-based antibiotic educational games for children.

**Figure 1:**
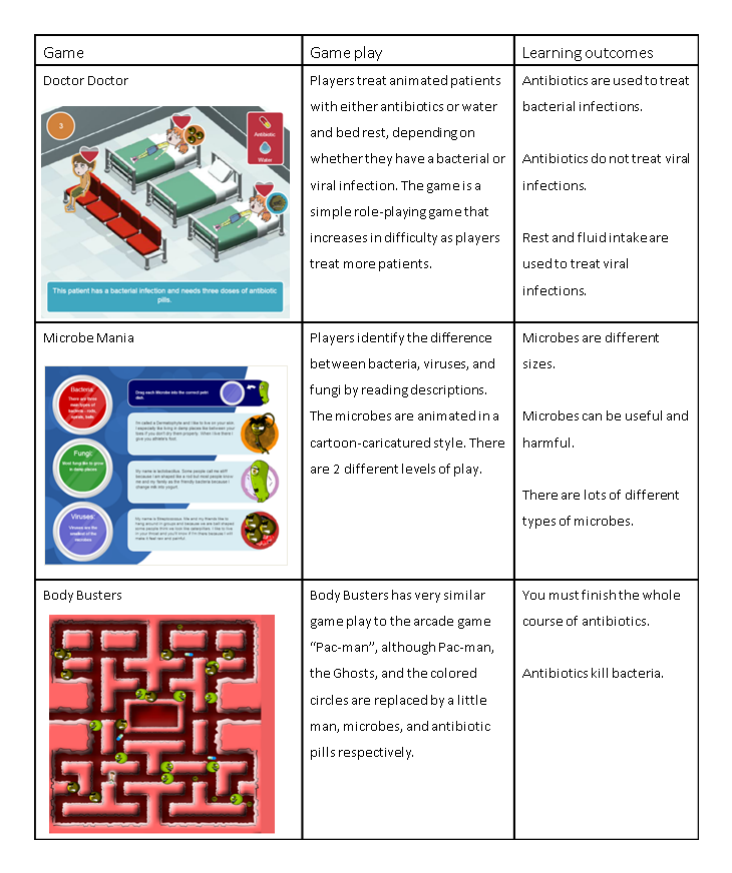
The three 3 e-Bug games evaluated in this study.

We aimed to determine the effectiveness, flow, and value of the e-Bug junior games as a resource for increasing antibiotic knowledge and awareness in primary school pupils and receive their suggestions for improvement. We selected 3 e-Bug games for this study based on their learning outcomes on antibiotic use, antibiotic resistance, and viruses or bacteria. The games are summarized in Figure 1. The aim of the e-Bug website is that children play the games in a classroom or at home, therefore, we evaluated the effect of playing all 3 games in sequence.

## Methods

### Setting

The authors invited, by email and telephone, 53 primary schools and 3 residential summer holiday schools in the Bristol and Gloucestershire area, which had not previously used the e-Bug resources in this setting, to participate. No incentives were offered.

### Recruitment

Three schools that expressed initial interest were sent detailed information. This included a parental information sheet with an opt-out form attached, a teacher consent form, and examples of the questionnaire, focus group and think-aloud material to be used. Schools that expressed no interest were not recontacted. Two residential summer and 2 primary schools withdrew as the timing of the study did not fit their lesson schedule.

#### Sample Size

A convenience sample of 153 pupils was used from the school and summer school that responded to the invitation to participate in the study.

#### Ethics

Ethics approval was gained from the University of the West of England Faculty Research Ethics Committee (UWE FREC). Written consent was obtained from teachers at the participating schools, and the parents and pupils involved were given the option for pupils to opt out at any point of the data collection.

All year, 5 and 6 pupils aged 9-11 years at the primary school and all pupils aged 9-11 years at the summer holiday school had the opportunity to evaluate the games. All data collection was carried out under the supervision of teachers in computer suites in the schools. The research was carried out in groups of around 30 pupils at a time. Before and after spending 15 minutes playing the 3 games, the groups of pupils completed questionnaires in silence, to ensure no comparison of answers. They played each of the 3 games (Doctor Doctor, Microbe Mania, and Body Busters) for 5 minutes, always in that order to maintain consistency across the schools. Time was also monitored to maintain consistency. During the game play time, pupils were allowed to talk freely, which allowed the researcher to gather verbal feedback on the games.

### Quantitative Data Collection

**Figure 2:**
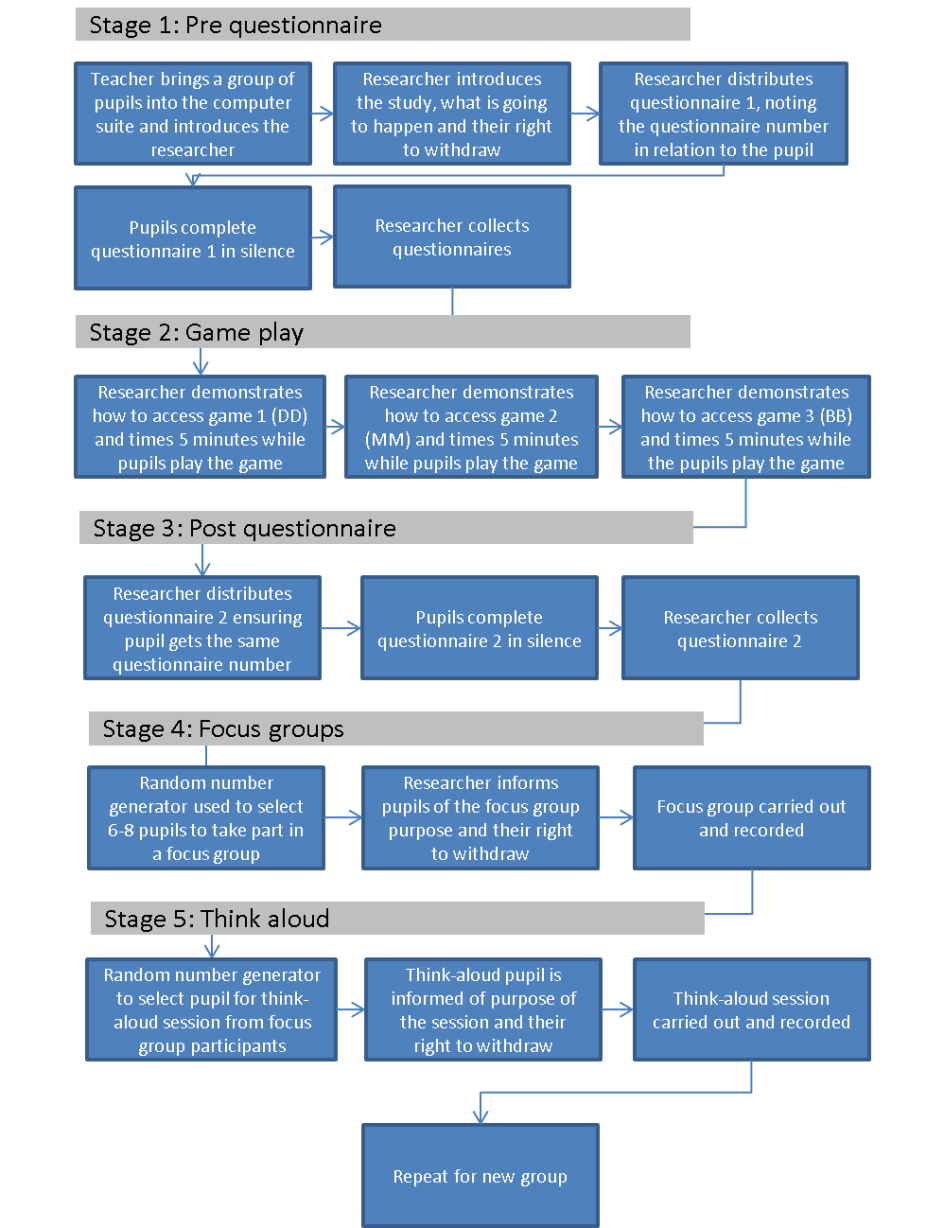
Data collection process.

The data collection process is outlined in Figure 2. The pregame questionnaire asked 7 simple multiple-choice questions adapted from a previous e-Bug evaluation [22]. Pupils’ responses were recorded as right or wrong. The answers to the questions were either available directly from information in the games or implied by in-game action (Multimedia Appendix 1) *.* The postgame questionnaire had identical questions. The questionnaires were matched to allow evaluation of knowledge change in individual pupils. The postgame questionnaire also asked how much the pupils liked each game, using a 10-point Likert scale and open-ended free text questions.

All incomplete answers in section 1 of the questionnaire were marked as wrong for data analysis. Questions with more than 1 answer ticked and incomplete answers in section 2 of the questionnaire were not included in the data analysis. We used a McNemar test to compare the pre- and postgame questionnaire answers, as this can compare 2 paired dichotomous variables. As statistical package for the social science (SPSS) version 20 does not provide a McNemar test without the Yates Correction (a correction for continuity used in the McNemar test that is often considered to be too conservative [23]), we employed a macro written by Marcia Garcia-Granero to remove the Yates correction [24].

### Qualitative Data Collection

After questionnaire completion, a random number generator was used to select 6 to 8 pupils to take part in a focus group at each venue. The focus group aimed to explore the usability of the games section of the website and assess the pupils’ change in awareness of antibiotics. Before participation, pupils were informed of the purpose of the focus group, that it would be recorded, and that they could opt out at any point. The focus group guide asked simple open-ended questions relating to the pupils’ thoughts on the games. Each focus group lasted at least ten minutes.

A think-aloud session followed the focus group. One pupil was selected randomly from the focus group at each venue and asked to describe their thoughts and feelings as they played the games. The researcher prompted the pupil to continue talking at all times and aimed to make each think-aloud session last at least five minutes. Throughout all data collection, the researcher recorded general observations and comments relating to the games in note form.

The researcher made verbatim transcripts of each session and the data were analyzed using NVivo qualitative analysis software (QSR International). Comments and interactions were marked as either positive or negative toward an aspect of the games and further divided into subgroups that related to comments about usability, style, learning experiences, or general comments.

## Results

### Recruitment

Ninety-three pupils aged 9-11 years were recruited from one primary school in the Bristol area and 60 pupils aged 9-11 years were recruited from a music-based summer school in Gloucestershire. In total, 153 pupils completed pre- and postgame questionnaires; 135 pupils completed the qualitative section of the post questionnaire. Forty-eight pupils completed 6 focus groups (3 groups in each venue with 8 pupils in each group) and 4 pupils (1 from the primary school and 3 from the summer school) participated in think-aloud sessions.

### Likert Scale Data

**Figure 3:**
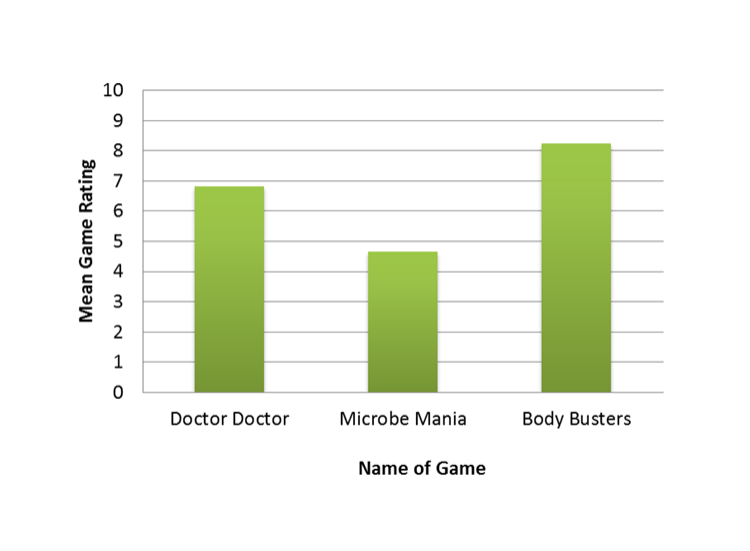
Comparison of the mean score given for each game in section 2 of the questionnaire.

**Figure 4:**
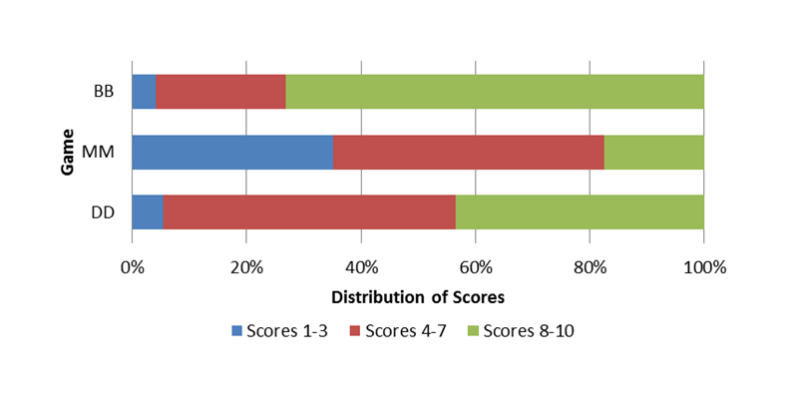
Comparison of the distribution of scores between the 3 games.

Body Busters had the highest mean score of 8.2 on the Likert scale, for how much participants reported enjoying playing the game (Figure 3). Microbe Mania had a mean score of 4.7, and Doctor Doctor had a mean score of 6.8. Distribution of the scores for each game (Figure 4) shows that 73.1% (106/145) of the scores for Body Busters were 8 to 10 whereas the scores for Microbe Mania were more evenly distributed across 1-10, with only 18% (26/148) scoring 8-10. Scores for Doctor Doctor were mostly distributed between scores 4-7 and 8-10 with only 5.4% (8/147) scoring 1-3.

### Qualitative Data

#### Doctor Doctor

Doctor Doctor received an overall positive review with many positive comments as well as suggestions for improvement.

I thought it was really fun in the fact that it was hard because there were 4 patients and only 3 beds

Student, focus group 4

Participants in the focus groups often agreed with comments made by other participants, with multiple pupils offering simple statements of agreement whenever a positive or negative statement was made. There were some negative comments surrounding the game play, including comments on how hard the game was.

I found it hard because I didn’t really understand too well, you had to explore the controls for yourself, because the instructions aren’t that good.

Student, focus group 4

I think it was actually quite frustrating too because when they were about to die you couldn’t really cure them at all and it was really really bad.

Student, focus group 5

There were also several suggestions to extend the game to incorporate more role-playing, integrating more of a story and characterization to increase immersion.

Instead of them dying you could like have another level that they go into like an emergency area instead of them dying.

Student, focus group 6

The learning experience of Doctor Doctor was well received. Many of the pupils demonstrated awareness of the topics covered during the gameplay.

If you got a virus then you’d know to give the patient water and rest and if they had a bacteria then you should give them antibiotics.

Student, focus group 6

No participants in the think-aloud sessions or free text section of the questionnaires made any negative comments regarding the learning experience. The small number of negative comments in the focus groups mostly came from pupils who admitted that they did not read the introductory information on how to play. One pupil mentioned that they thought the colors in the game worked really well together. Although the pupils mostly liked the characters, they expressed a wish for more variation.

#### Microbe Mania

Microbe Mania received an overall negative review, with many negative comments and no positive comments regarding the in-game action. Common themes from the focus groups were that the game did not have many “gaming” elements and it was more like a quiz than a game.

It wasn’t a game, it was just information.

Student, focus group 2

When we had to play microbe mania and it was the same game over and over again.

Q 12, participant 40

The pupils also said that the main problem was that the game needed expansion beyond the 2 short levels available, and some element of challenge. The pupils suggested the game could be improved by adding a time limit and having more and varied levels. Some of the comments regarding the lack of challenge stem from the game’s mechanics that allow a player to guess the answers without repercussions, taking away the need to read any of the information, a factor that was noted by the pupils and increased some of the negative opinions of the games.

It also lets you do it as many times as you want to, you could literally just go for every one.

Student, focus group 2

The microbe mania needs to be longer and less boring.

Q 13, participant 130

The apparently poor gameplay of Microbe Mania meant the pupils lacked interest, offering many comments of “boring” in the focus groups. Microbe Mania was consistently commented on as the worst game in the free text section of the questionnaires. This correlates with observations in the researcher’s field notes, that showed that the pupils very quickly became bored with this game and wanted to move on to a more interesting one. Their lack of enjoyment affected the learning experience that accompanied the game; almost no comments showed a positive learning experience in any of the data collection methods. In some brief instances pupils demonstrated the knowledge available in the game but many said that they just guessed the answers.

#### Body Busters

Body Busters was the most popular of the 3 games played by the pupils. When asked, the pupils consistently said that this was their favorite game. The free text questionnaire section also showed that Body Busters was very popular, with a large number of pupils writing that it was their favorite section.

What I like about the game is that the more you play it and you collect the more pills the less, the smaller amount of bacteria and viruses, which makes it easier.

Think-aloud 2

Yeah that was so cool, the main ones didn’t go away so it was fun and challenging.

Student, focus group 4

My favourite was when we had to run away from the germs.

Q 11, participant 112

The few negative comments about the game mostly arose from not knowing how to play but such comments were very rare in comparison to positive comments.

It didn’t really explain, I don’t know if there was a how to play on the advert, I didn’t see it.

Student, focus group 2

Overall, a generally positive learning experience appeared to accompany the game, although when asked what they had learnt, none of the comments were linked to the learning objectives (“you must finish the whole course of your antibiotics,” and “antibiotics kill bacteria”). This was the only game that prompted further discussion directly related to any of the subjects addressed. Although the other games prompted some antibiotic or microbe-related questions, Body Busters prompted a brief discussion (below) that showed the beginning of some change in awareness about antibiotic use.

P: I thought pills were bad for you.

R: You thought pills were bad for you?

P: Because sometimes people die from having pills.

R: Those are bad pills though, there are lots of different sorts.

P: Aren’t they drugs, the pills?

R: Antibiotics? Pills just hold things; they can hold lots of different things so some are good for you and some are bad for you. These are antibiotics and are good at killing bad bacteria, so the ones that make you ill.

P: There are good bacteria as well.

R: There are good bacteria as well.

Focus group 3, P: participant, R: researcher

Several comments suggested improvements to the game, including improvement to the overall design.

Maybe for the pacmanish game it could have more characteristics for the viruses and stuff.

Student, focus group 1

*And maybe more lives so you don’t die as soon as you touch them.* [Student, focus group 1]

### Quantitative Data

After playing, there was a small increase in the number of participants answering each question correctly (Multimedia Appendix 1). This increase ranged from 2.0% for the question, “Which of these microbes causes coughs and colds” to a 13.1% increase for the question, “Which of these would antibiotics be used for?” However, the increase was only significant for the 2 questions (5 and 7) which tested pupil’s knowledge about the effectiveness of antibiotics against bacteria and viruses (*P*<.05). In question 5, 26.8% pupils (41/153) answered incorrectly before and correctly after playing the games; in question 7, 15% pupils (23/153) answered incorrectly before and correctly after playing the games.

The highest knowledge in the prequestionnaire came in the question “most coughs and colds get better without antibiotics,” with 68.6% (105/153) of pupils answering correctly. The lowest knowledge in the prequestionnaire was in question 7, which focused around what antibiotics do. Only 9.2% (14/153) pupils answered this correctly, although this saw one of the largest increases in knowledge in the postquestionnaire.

## Discussion

### Principal Findings

This study indicates that playing the 3 games consecutively in one session had a small significant effect on pupils’ knowledge of antibiotics. Body Busters, which teaches that antibiotics kill bacteria and that you must finish your whole course of antibiotics, was the most effective game for generating discussion about and increasing awareness of antibiotics. It promoted the most positive discussion about flow and enjoyment in the focus groups and ranked the highest on the Likert scales. This suggests that enjoyment of a game is an important factor in learning and in the amount of awareness a game is able to impart. Data from Doctor Doctor supports this suggestion; it received generally positive reviews but was not as successful at changing knowledge as Body Busters. None of the questions that linked to Microbe Mania had any significant change in knowledge, and qualitative data showed that it was neither popular, interesting, nor a good learning experience. This accords with Csikszentmihalyi’s theory of flow, that suggests that if a player is enjoying a game then they will become more engaged with it and take in more of the available information [11-13]. The data gathered in relation to Body Busters could demonstrate that it creates a flow-like state in players, whereas Microbe Mania does not.

### Strengths and Limitations

The strengths of this study were that the data were collected in a school environment, emulating the environment where the games would normally be played as part of structured e-Bug lessons. This removes distractions that would come from carrying out the study in an environment unfamiliar to the pupils. Another strength is that the games were played together, simulating what a pupil may do during unstructured use. Finally, antibiotics are not covered in the national curriculum at this age, allowing a more accurate reflection of knowledge change due to game play.

All postgame data collection was done immediately after playing the games, therefore we do not know if the increased awareness and knowledge was either maintained or changed future behavior. The choice to collect data immediately after the pupils played the games was governed by the time constraints prevailing on the researcher and the teachers and educators at the institutions where the research was carried out.

Another limitation was that the think-aloud methodology is not well suited to being used with young pupils. The pupils aged 9-11 years who participated in this study often found it difficult to vocalize their thoughts beyond simple sentences or reading directly from the screen. The pupils’ poor responses may have been due to the small sample size and chance, as the pupils were chosen at random from the group. Responses from the pupils may have been affected by the type of questions; developing simple multiple choice questions relating a complex topic such as antibiotic resistance can limit or bias the answers.

The evaluation was done after the games were played for 15 minutes in isolation, which may not mimic the natural environment for game play. The games are likely to be more effective when used to reinforce teaching in the classroom, alongside the curriculum, but more work will be needed to confirm this. A further study could ask pupils to play the games several times, to see if knowledge increases over time. The similar questions in the pre- and postgame questionnaires may cause the pupil to learn through the questionnaire rather than the game. Varying the questions asked while assessing the same learning outcomes could address this issue.

### Implications

The Body Busters game was shown to be a good tool for changing awareness of antibiotics. Particular elements of the game that contribute to flow, user engagement, and enjoyment include the colorful and exciting visuals, the simple, relatable and easily understood gameplay, and the fast-paced action. Pupils suggested very few improvements other than overall expansion. An increase in knowledge on the learning outcomes could be supported by including more information in the introductory text, making the difference between viruses and bacteria more obvious, and creating a steady increase in difficulty as the game progresses.

The Doctor Doctor game is also well suited to its role in e-Bug, showing an increase in awareness for its learning objective (antibiotics are used to treat bacterial infections). Similar to Body Busters, this game uses exciting and colorful visuals and simple gameplay to encourage flow and user engagement, as well as other successful elements such as a strong narrative to “save the patients,” and progressively harder and more challenging gameplay. A further increase in knowledge for the learning outcomes could be attained by lengthening the game, either with further levels using different scenarios, or a wider variety of difficulties. If further levels are included, asking the player to answer questions between levels, with in-game rewards, would benefit both knowledge on the leaning objectives and the flow.

Microbe Mania would benefit from more significant design and gameplay changes, as it offers no benefit to the website and may even detract from the overall purpose of e-Bug. Although there are a few previously identified elements of flow in this game, such as a colorful style, they do not come together to form any sort of user engagement. Increasing the overall engagement and flow could be achieved through the use of rewards for the correct answer, penalties for incorrect answers, and/or time limits for completing each level. Other improvements include increasing the text size, a much broader range of questions and answers, more levels, and a quiz at the end of the game. Although Microbe Mania has the potential to be more of an asset to the e-Bug website, feedback from teachers suggests that it should be moved to the teacher-led section of the website, as teachers find the information useful to reinforce learning outcomes in the microbes’ lessons.

This study could stand as a basis for a much larger study identifying the role of educational games as teaching resources and as a broader evaluation of the e-Bug material. The e-Bug project will hopefully continue to increase awareness of antibiotics in Europe and help reduce antibiotic use, thereby reducing the rise in superbugs.

## Appendix

Multimedia Appendix 1 Questions and answers, where answers were available in game, and percentage of pupils that answered correctly before and after playing the games for each question (N=153).
