# Supplementary material for: Can Gaming Increase Antibiotic Awareness in Children? A Mixed-Methods Approach
Source: JMIR Serious Games. 2017 Mar 24;5(1):e5. doi: 10.2196/games.6420 (PMC5384992; doi:10.2196/games.6420)
Supplement: Multimedia Appendix 1 [file games_v5i1e5_app1.pdf]

Question no.	1	2	3	4	5	6	7
Question	Which of these microbes causes coughs and colds?	What's the best way to kill a virus?	Can antibiotics help cure a cold?	Microbes can help make...	Which of these would antibiotics be used for?	Most coughs and colds get better without antibiotics	Finish the sentence (circle one) Antibiotics...
Possible answers	Bacteria, fungus, virus, Prion	Eating lots, water and bed rest, antibiotics, exercise	Yes, no, sometimes	Onions, cheese, coffee, chocolate	Bacteria, virus, badly scraped knees	True, false	Are good at killing viruses, are bad for you, can make you smarter, only work if you finish the course
Correct answer	Virus	Water and bed rest	No	Cheese	Bacteria	True	Only work if you finish the course
Associated game	Microbe Mania	Doctor Doctor	Body Busters or Microbe Mania	Microbe Mania	Body Busters or Microbe Mania	Doctor Doctor	Body Busters
Where answer to question is available in the games	Information can be read in text boxes available in-game	Information available from text box in the game and from the in-game action	Information in text boxes available in Microbe Mania and introductory text box in Body Busters as well as through gameplay	Information can be read in text boxes available in-game	Links to in-game action and text boxes in Doctor Doctor and the text box and in-game action of Body Busters	Links to in-game action	Links to in-game action
McNemar χ2	0.21	2.12	1.2	3.27	6.45	0.56	11.57
P value	.65	.15	.27	.71	.01	.46	<.001
95% CI of the % increase in correct answers	−10.41 to 6.52	−16.67 to 2.50	−11.06 to 3.22	−14.82 to 0.62	−22.75 to −3.02	−11.86 to 5.39	−18.68 to −5.12
n (%) correct before	40 (26.1)	57 (37.2)	33 (21.6)	70 (45.8)	42 (27.5)	105 (68.6)	14 (9.2)
n (%) correct after	43 (28.1)	68 (44.4)	40 (26.1)	81 (52.9)	62 (40.5)	110 (71.9)	32 (20.9)
n (%) with improved score	23 (15.0)	34 (22.2)	19 (12.4)	24 (15.7)	41 (26.8)	25 (16.3)	23 (15.0)
n (%) correct in both questionnaire	20 (13.1)	34 (22.2)	21 (13.7)	57 (37.3)	21 (13.7)	85 (55.6)	9 (5.9)
n (%) incorrect in both questionnaires	90 (58.8)	62 (40.5)	101 (66.0)	59 (38.6)	70 (45.8)	23 (15.0)	116 (75.8)
% increase in correct answers	2.0	7.2	4.6	7.19	13.1	3.3	11.8

Table 1. Questions and answers, where answers were available in game, and percentage of pupils that answered correctly before and after playing the games for each question (N=153).
